# Attribution of colorectal cancer symptoms to medications for pre-existing chronic conditions: a secondary analysis of a vignette study in England

**DOI:** 10.1093/pubmed/fdaf014

**Published:** 2025-02-20

**Authors:** Giovanni E Ricciardi, Flavia Pennisi, Christian Von Wagner, Lauren Smith, Aradhna Kaushal, Gary A Abel, Georgios Lyratzopoulos, Cristina Renzi

**Affiliations:** PhD National Programme in One Health Approaches to Infectious Diseases and Life Science Research, Department of Public Health, Experimental and Forensic Medicine, University of Pavia, Pavia 27100, Italy; School of Medicine, Università Vita-Salute San Raffaele, via Olgettina, 58, 20132, Milano, Italy; PhD National Programme in One Health Approaches to Infectious Diseases and Life Science Research, Department of Public Health, Experimental and Forensic Medicine, University of Pavia, Pavia 27100, Italy; School of Medicine, Università Vita-Salute San Raffaele, via Olgettina, 58, 20132, Milano, Italy; Epidemiology of Cancer Healthcare & Outcomes (ECHO), Research Department of Behavioural Science and Health, Institute of Epidemiology and Health Care (IEHC), University College London, London, UK; Epidemiology of Cancer Healthcare & Outcomes (ECHO), Research Department of Behavioural Science and Health, Institute of Epidemiology and Health Care (IEHC), University College London, London, UK; Epidemiology of Cancer Healthcare & Outcomes (ECHO), Research Department of Behavioural Science and Health, Institute of Epidemiology and Health Care (IEHC), University College London, London, UK; University of Exeter Medical School, Department of Health and Community Sciences, Faculty of Health and Life Sciences, University of Exeter, St Luke’s Campus, Heavitree Road, Exeter EX1 2LU UK; Epidemiology of Cancer Healthcare & Outcomes (ECHO), Research Department of Behavioural Science and Health, Institute of Epidemiology and Health Care (IEHC), University College London, London, UK; School of Medicine, Università Vita-Salute San Raffaele, via Olgettina, 58, 20132, Milano, Italy; Epidemiology of Cancer Healthcare & Outcomes (ECHO), Research Department of Behavioural Science and Health, Institute of Epidemiology and Health Care (IEHC), University College London, London, UK

**Keywords:** chronic conditions, colorectal cancer, diagnostic delays, symptom attribution, therapy

## Abstract

**Objective:**

To investigate the likelihood of attributing colorectal cancer (CRC) symptoms to medications for chronic conditions.

**Methods:**

The online vignette survey included 1287 participants aged ≥50 years, with quota sampling to recruit sufficient participants with type 2 diabetes. Participants self-reported chronic conditions and answered questions on symptom attribution and help-seeking, after reading vignettes describing new-onset rectal bleeding or change in bowel habit. Using multivariable logistic regression, we analyzed the association between specific conditions and attributing new-onset CRC symptoms to medications, controlling for demographics.

**Results:**

Among participants, 25% reported type 2 diabetes, 31% being overweight, 25% hypertension and 22% arthritis. Participants with diabetes, versus those without, had a higher likelihood of attributing change in bowel habit to medications [7% vs 3%; adjusted Odds Ratio (aOR) 2.55, Confidence Interval (95% CI) 1.30–5.00]. This was also the case for participants reporting being overweight (7% vs 2%; aOR 2.36, 95% CI 1.25–4.44), arthritis (8% vs 3%; aOR 2.27, 95% CI 1.19–4.35), but not for hypertension. No significant association was found regarding attribution of rectal bleeding to medications.

**Conclusions:**

Patients with common chronic conditions have a higher likelihood of attributing change in bowel habit to medications. Tailored information is needed for these patients, encouraging them to discuss any new symptom with their doctor.

## Introduction

Colorectal cancer (CRC) is the fourth most common cancer in the UK, with the second highest cancer-related mortality.^1^ Despite progress in screening and early diagnosis, almost half of CRCs are still diagnosed at an advanced stage,^2^ reducing treatment options and survival. Between 70% and 95% of CRC are diagnosed after patients present with typical red flag symptoms (rectal bleeding or change in bowel habit), or general symptoms (unexplained weight loss or fatigue).^3^ This highlights the importance of patient symptom appraisal, as a pre-requisite for timely help-seeking and cancer diagnosis.^4^ However, the recognition of cancer symptoms can be particularly complicated for patients with pre-existing chronic conditions,^5^^,^^6^ as some diseases and their pharmacological treatments can be associated with symptoms overlapping with those of cancer.^7^^,^^8^ This is especially concerning given the increasing prevalence of multimorbidity in older adults, the age-group also affected by a higher cancer risk.^9^ While various studies investigated the association between chronic conditions and cancer symptom attribution, evidence on medications as possible alternative explanations is scant.^6^^,^^10^

The study aimed to examine the likelihood of attributing new-onset CRC symptoms to medications in people with common chronic conditions.

## Methods

We conducted an online vignette survey in the UK using the online platform Prolific, recruiting people aged 50+ years with no recent cancer diagnosis. Employing quota sampling, we recruited 25% of the sample with type 2 diabetes to ensure a sufficient number of participants with this disease, which was considered of particular interest based on previous research.^11^ This study is part of a wider project using online vignettes to investigate the role of chronic morbidities in influencing the diagnosis of cancer. Study details have been previously described.^6^ Briefly, participants were asked to report any chronic condition they were ever diagnosed with, selecting them from a pre-coded standard list. Subsequently, participants were invited to read the same two vignettes describing new-onset symptom, in particular one vignette describing rectal bleeding and the other vignette describing change in bowel habit, followed by questions on symptom attribution and intended help-seeking. Using multivariable logistic regression, we analyzed the association between the presence of specific conditions and the likelihood of attributing new-onset change in bowel habit or rectal bleeding to medications, controlling for sex, age, ethnicity, and history of stool tests and colonoscopy. All conditions were included in the same multivariable model, but each of the two symptoms was examined in a separate model. The study was approved by the UCL Ethics Committee (N14687/006).^6^

## Results

The study included 1287 participants, with 61% being women, 62% aged 50–59 and 87% reporting white ethnicity. The most frequent chronic conditions included type 2 diabetes (25%), obesity (31%), hypertension (25%), and arthritis (22%), (Table 1). Overall, 28% of participants had previously undergone a colonoscopy or sigmoidoscopy, and 55% had a stool test history.

**Table 1:** Characteristics of participants and prevalence of chronic conditions in the study sample.

	Total
	N = 1287
**Age**	
50–59	791 (61.5%)
60–69	399 (31.0%)
70+	97 (7.5%)
**Sex**	
Male	500 (38.9)
Female	782 (60.8%)
Prefer not to say^a^	5 (0.4%)
**Ethnic Group**	
White	1123 (87.3%)
Other	164 (12.7%)
**Past colonoscopy/sigmoidoscopy**	
Yes, for screening	101 (7.9%)
Yes, for symptoms	260 (20.2%)
No	926 (72.0%)
**Past Stool test**	
Yes, for screening	475 (36.9%)
Yes, for symptoms	226 (17.6%)
No	586 (45.5%)
**Chronic conditions**	
Overweight	349 (30.6%)
Diabetes Type 2	320 (24.9%)
Hypertension	317 (24.6%)
Arthritis	284 (22.1%)
Mental Health problems	183 (14.2%)
Back problems	169 (13.1%)
Asthma	159 (12.4%)
Irritable bowel syndrome (IBS)	102 (7.9%)
Haemorroids	80 (6.2%)
Heart problems	65 (5.1%)
Diverticular	48 (3.7%)
Deafness	33 (2.6%)
Kidney and Liver diseases	30 (2.3%)
Chronic obstructive pulmonary disease (COPD)	26 (2.0%)
Neurological problems	22 (1.7%)
Endometriosis	14 (1.1%)
Diabetes Type 1	11 (0.9%)
Epilepsy	11 (0.9%)
Blindness	8 (0.6%)
Alzheimer or dementia	0 (0.0%)
Other	127 (9.9%)
**Two or more chronic conditions**	632 (49.1%)

a The group was removed from further analyses due to small numbers.

**Figure 1:**
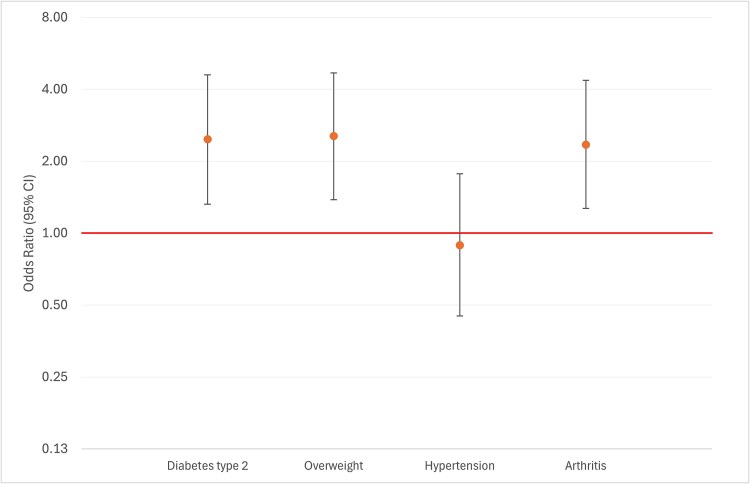
Attribution of change in bowel habit to medications by participants with type 2 diabetes, overweight, hypertension, and arthritis: Multivariable logistic regression odds ratios, adjusted for age, gender, ethnicity, comorbidity number, and previous investigations. The y-axis is presented on a logarithmic scale for odds ratios.

Participants with type 2 diabetes were much more likely to attribute change in bowel habit to medications, compared to participants without diabetes [7% vs 3%; adjusted Odds Ratio (aOR) 2.55, Confidence Interval (95% CI) 1.30–5.00], as were participants with obesity (7% vs 2%; aOR 2.36, 95% CI 1.25–4.44) and arthritis (8% vs 3%; aOR 2.27, 95% CI 1.19–4.35) (Fig. 1). On the contrary, we did not find a significant association between hypertension and attributing change in bowel habit to medications (4% vs 4%; aOR 0.65, 95% CI 0.23–1.35). No significant associations emerged regarding the attribution of rectal blending to medications for any of the examined conditions.

## Discussion

Patients with diabetes, compared to those without, have a two-fold higher likelihood of attributing change in bowel habit, a typical red-flag CRC symptom, to medications. This was also the case for individuals with obesity or arthritis. Gastrointestinal side effects such as change in bowel habit are commonly associated with metformin and glucagon-like peptide-1 receptor agonists (GLP-1RAs), both of which are primary treatments for type 2 diabetes.^12^ Similarly, GLP-1RAs are increasingly prescribed for weight loss in individuals with obesity.^13^ This can explain our study findings indicating a higher likelihood of attributing CRC symptoms to medication side effects in respondents with diabetes or obesity​. Non-steroidal anti-inflammatory drugs (NSAIDs), commonly used for osteoarthritis and rheumatoid arthritis, can also lead to gastrointestinal symptoms.^14^ The tendency to attribute symptoms to possible side effects of chronic disease treatments rather than to a possible as-yet undiagnosed cancer aligns with the “alternative explanation” mechanism.^11^ Our previous study focusing on type 2 diabetes found that diabetic patients have a lower likelihood of attributing CRC symptoms to cancer, more frequently think it might be caused by medications, and when seeing a doctor they prioritize concerns related to their condition rather than discussing typical red-flag CRC symptoms.^6^ In the current study, we expanded the analyses beyond diabetes including also other conditions. This highlighted how attributing cancer symptoms to medications is a relevant mechanism also in patients with other common conditions. While previous studies have examined the association between chronic diseases and cancer symptom attribution and impact on diagnostic delays,^15^^,^^16^ evidence on medications as possible alternative explanations is scant.^6^^,^^15^^,^^16^

We recognize several limitations in our findings. Firstly, in this study we did not have data on the specific treatments each patient was receiving, and this should be addressed in future research. While we controlled for factors such as gender, age, race, and history of fecal testing and colonoscopy, other variables, including lifestyle factors, diet, genetic factors, or family history, could influence symptom attribution. Future studies could explore these additional variables to gain a more comprehensive understanding of the issue. Furthermore, the cross-sectional design of this study limits our ability to determine causal relationships. Longitudinal studies could better investigate changes in health behaviors, symptom attribution and cancer diagnosis over time in patients with chronic diseases. Additionally, further research based on interdisciplinary collaborations could provide a deeper understanding of the complex interplay between clinical, pharmacological, psychological, and sociological factors in influencing cancer symptom attribution and health behaviors. Our study used multivariable logistic regression, but future research could also benefit from more complex statistical approaches like multilevel models or structural equation modeling. Finally, cultural and socio-economic factors should be taken into account when considering the generalizability and relevance of the findings in different contexts. The study findings emphasize the need to raise awareness among patients with common chronic conditions regarding the potential overlap between symptoms related to medications for chronic diseases and potential cancer symptoms. Patients should be encouraged to discuss any new or unusual symptoms promptly with their healthcare provider. Targeted educational intervention could help these patients become better equipped to recognize and manage potential cancer symptoms. Appropriate communication is needed, providing patients with balanced information to avoid causing unnecessary worry or over-burdening the health system. Research is needed on appropriate doctor-patient communication strategies, especially for older patients with multimorbidity.^17^ Additionally, risk stratification approaches and tests that can aid the differentiation between possible medication side effects and symptoms of an as-yet undiagnosed cancer are essential to support early cancer diagnosis for the increasing number of older multimorbid patients.

## Data Availability

The data supporting the findings of this study can be made available upon reasonable request from the corresponding author.

## Notes

**Giovanni E. Ricciardi**, Dr

**Flavia Pennisi**, Dr

**Christian Von Wagner**, Prof

**Lauren Smith**, Dr

**Aradhna Kaushal**, Dr

**Gary A. Abel**, Dr

**Georgios Lyratzopoulos**, Prof

**Cristina Renzi**, Prof
